# Five factors contributing to severe rhabdomyolysis in a 21 yr old IV drug abuser: a case report

**DOI:** 10.4076/1757-1626-2-6479

**Published:** 2009-07-07

**Authors:** Prasanthi Ganeshram, Poorani Nallam Goundan, Vijay Jeyachandran, Preetham Arthur

**Affiliations:** Department of General Medicine, Sri Ramachandra UniversityChennai, Tamil NaduIndia

## Abstract

Rhabdomyolysis is a potentially life-threatening condition resulting from the release of large quantities of myocyte breakdown products into the circulation, following injury to striated muscles. There are several causes of rhabdomyolysis - traumatic and non-traumatic. We present a 21-year-old male intravenous drug abuser, who was referred to us with fever, altered sensorium and seizures. He developed severe rhabdomyolysis following a mixed meningeal infection by *Streptococcus pneumoniae* and *Mycobacterium tuberculosis*. This patient’s examination and investigation suggested a combination of factors leading to the severe rhabdomyolysis which proved fatal. The patient’s creatine phosphokinase was elevated to 167,000 U/L, following hyperpyrexia, seizures, meningitis (pneumococcal and tuberculous), pentazocine and alcohol abuse. The increase in mortality rate with the onset of rhabdomyolysis warrants immediate cessation of the insult and aggressive management.

## Introduction

Rhabdomyolysis (RM) is a syndrome characterised by muscle breakdown and necrosis, resulting in elevated serum concentration of creatine kinase (CK) and myoglobinuria [1]. It could be generalized, or may involve specific groups of muscles [2]. The classic triad of symptoms includes weakness, tea-coloured urine and muscle pain. The diagnosis requires a certain amount of clinical suspicion as it can be asymptomatic. Life threatening complications such as acute renal failure, cardiac arrhythmias, cardiac arrest, hyperkalemia, hypocalcemia and disseminated intravascular coagulation can occur, thereby requiring aggressive management [3].

## Case presentation

A 21-year-old Indian male was referred to our tertiary care centre with a history of high grade fever, chills and rigors for 10 days. He was found to have altered sensorium for 4 days and multiple episodes of partial tonic clonic seizures of the left lower limb for a day. There was no history of vomiting or development of rashes. He had received treatment for fever and altered sensorium in another hospital. The details of the treatment were not known. He was a known IV abuser of pentazocine and promethazine for over 4 years. The last dose was taken 10 days prior to admission. The patient is a known alcoholic and smoker for about 4-5 years, but the details of the quantity abused is not known. He was not a known case of hypertension, epilepsy, tuberculosis, diabetes or psychiatric illness. He was unmarried. Sexual history could not be assessed as the patient had altered sensorium. His relatives denied any known sexual promiscuity.

On examination, the patient was conscious and disoriented with a Glasgow coma scale of 8/15 (E3, V2, M3). He was febrile, with a temperature of 106 F. Vital signs showed a pulse rate of 120 beats/min, BP of 130/90 mmHg, and respiratory rate of 22/min. Central nervous system examination elicited nuchal rigidity and decreased power in the left lower limb (1/5). Papilledema was identified on optic fundus examination. All other systems examination was unremarkable. There were no obvious rashes seen.

Investigations showed leucocytosis with neutrophilic preponderance and a normal platelet count. Apart from an elevated AST, renal and liver function tests were normal (Table 1). CSF analysis was suggestive of lymphocytic predominant meningitis (Table 1). Blood smear was negative for malarial parasite. Imaging of the brain by CT scan showed cerebral edema with no focal lesion or hydrocephalous. Urine analysis was positive for proteinuria (1+). A provisional diagnosis of partially treated meningoencephalitis was made and the patient was empirically started on acyclovir, ceftriaxone and supportive measures. Further microbiological analysis of the CSF was negative for HSV, but a PCR for *Mycobacterium tuberculosis* was positive. CSF culture grew *Streptococcus pneumoniae* sensitive to piperacillin and tazobactum. Magnetic resonance imaging of the brain showed cerebral edema with no features of HSV encephalitis, and the chest x-ray was reported normal. Thereafter, acyclovir was stopped and the patient was started on piperacillin, tazobactum (after dose calculation as per GFR) and antituberculous drugs, namely, isoniazid (300 mg a day), rifampicin (600 mg a day), ethambutol (1200 mg a day), and pyrazinamide (1500 mg a day). The patient did not improve clinically. On the 4^th^ day of admission he continued to have a high grade fever. The urine was becoming high coloured and output was reaching the oliguric range. Serum creatine phosphokinase (CPK) and potassium were elevated to 167,000 U/L and 5.1 mEq/dl respectively, but the urine was negative for RBCs and myoglobin.

**Table 1.** Investigations

Laboratory investigations	Value
Total count	17,800 cells/ cu.mm
Differential count	P_87_, L_10_, E_3_, M_0_
Platelet count	2,75,000 / cu.mm.
AST	246 U/dL
Peripheral Smear	Normal RBCs, Leucocytosis with neutrophilic predominance, Adequate platelets. Negative for malarial and filarial parasites.
CSF Study:	106 mg/dl
Protein	73 mg/dl
Sugar	106 mg/dl
Chloride	100
Cells	70%
Lymphocytes	30%
Neutrophils	Negative
India ink	*Streptococcus pneumoniae*
CSF culture	
Blood culture	No growth
Urine culture	No growth
Serum creatinine	On admission On initiation of dialysis 0.9 mg/dl 6.1 mg/dl

**Figure 1.**
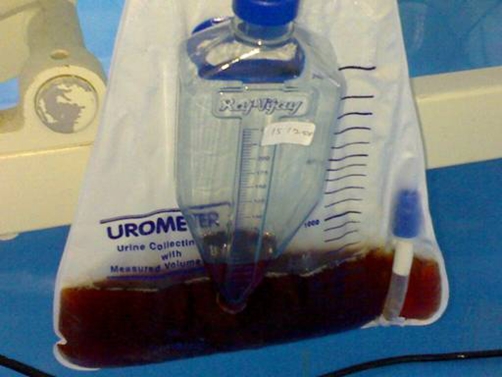
Urine appearance on day 6 of admission. Tea coloured urine characteristic of rhabdomyolysis.

Rhabdomyolysis was diagnosed and the patient was started on aggressive fluid therapy along with correction of hyperkalemia, alkaline diuresis and hemodialysis. The urine continued to be tea coloured (Figure 1) and on day 6, the patient developed cardiac arrest during dialysis. He was revived; however, the brain stem functions were lost. The dyselectrolytemia was refractory to treatment and the urine output did not improve, so hemodialysis was continued along with antimicrobials and fluid therapy. Patient suffered another cardiac arrest and died on day 9 of admission.

## Discussion

Rhabdomyolysis is commonly caused by alcohol abuse, muscle overexertion, muscle compression and the use of certain medications or illicit drugs [3]. Infectious etiology has been reported in about 5% of cases [1]. Muscle injury, either traumatic or non traumatic (i.e.: metabolic, toxin mediated, infectious) leads to the release of myocyte contents like myoglobin, electrolytes and enzymes. In an acidic pH, the myoglobin released obstructs the renal tubules and causes tubular necrosis; this eventually leads to ARF. Fluid sequestration by the injured muscle leads to hypovolemia and causes prerenal azotemia [4,5]. ARF complicates about 10 to 15% of cases [1,2], and is the most common complication. Our patient developed acute renal failure following rhabdomyolysis, which was caused by a combination of factors. The CPK was elevated to as high as 167,000 U/L. We believe the reason behind such high levels of CPK involved more than one cause. High grade fever not subsiding for more than 15 hours, partial seizures, pentazocine abuse, alcohol abuse and mixed meningeal infection, all contributed to the gross elevation of CPK and the severe rhabdomyolysis.

*Streptococcus pneumoniae* is a bacterium that is known to cause rhabdomyolysis. Though the pathophysiology is not clear, decreased glycolytic activity, toxins and direct invasion have been suggested [6,7]. Rhabdomyolysis occurring in pneumococcal infection increases the morbidity and mortality of the condition [8]. Though there are no reports of tuberculosis causing rhabdomyolysis, we suspect it could have been a contributing factor.

Our patient was an alcoholic and an abuser of pentazocine, a synthetic opioid. Alcohol abuse is a very common cause of rhabdomyolysis. Both short term intoxication and long term abuse have been reported to cause this disorder [9,10]. Opioids are a known cause of rhabdomyolysis, and pentazocine has been reported to cause rhabdomyolysis when taken along with alcohol [11]. This patient had partial seizures of his left lower limb and hyperpyrexia, which contributed to the severity of the disorder.

The diagnosis of rhabdomyolysis requires a certain degree of clinical suspicion as it can be asymptomatic. The patient usually presents with weakness, tea-coloured urine and muscle pain. Laboratory tests will show elevated serum CPK and myoglobin. Urine dipstick will be positive for blood without any RBCs and myoglobinuria. Our patient’s urine was negative for myoglobin. This did not rule out rhabdomyolysis as it is noted to be absent in about 50% of cases [12]. The elevated creatine phosphokinase to over 167,000 U/L with a background of pentazocine-alcohol abuse, seizures, hyperpyrexia and pneumococcal meningitis raised the suspicion of rhabdomyolysis.

Treatment consists of aggressive fluid therapy to replace the extracellular losses and also to aid in clearing myoglobin from the tubules. Initially, normal saline should be given at a rate of 1.5 L per hour. Urine output should be maintained at 300 mL per hour until myoglobinuria has ceased [12]. High rates of IV fluid administration should be used at least until the CK level decreases to or below 1,000 units per L [12]. Alkalization of urine to keep the pH above 6.5 helps in preventing tubular obstruction and clearing myoglobin from the tubules, as acidic urine can precipitate renal tubular obstruction by myoglobin [3]. Correction of hyperkalemia is mandated to prevent cardiac arrhythmias and cardiac arrest. Hemodialysis is warranted if acute tubular necrosis ensues, and must be continued on a daily basis [3].

## Conclusion

Rhabdomyolysis has several etiological factors and the knowledge of these causes raises the index of suspicion in recognising it, thus aiding early initiation of treatment. Aggressive management of rhabdomyolysis can prevent the dreaded complication of acute renal failure.

## Abbreviations

ARF: Acute renal failure
AST: Aspartate transferase
BP: Blood pressure
CK: Creatine kinase
CPK: Creatine phosphokinase
CSF: Cerebrospinal fluid
CT: Computed tomography
GCS: Glasgow coma scale
HSV: Herpes simplex virus
IV: Intravenous
PCR: Polymerase chain reaction
RBC: Red blood cell
RM: Rhabdomyolysis
TB: Tuberculosis
